# Long-Term Antiretroviral Treatment Adherence in HIV-Infected Adolescents and Adults in Uganda: A Qualitative Study

**DOI:** 10.1371/journal.pone.0167492

**Published:** 2016-11-29

**Authors:** Seth C. Inzaule, Raph L. Hamers, Cissy Kityo, Tobias F. Rinke de Wit, Maria Roura

**Affiliations:** 1 Dept of Global Health, Academic Medical Center of the University of Amsterdam, and Amsterdam Institute for Global Health and Development, Amsterdam, The Netherlands; 2 Dept of Internal Medicine, Div. of Infectious Diseases, Academic Medical Center of the University of Amsterdam, Amsterdam, the Netherlands; 3 Joint Clinical Research Centre, Kampala, Uganda; 4 ISGlobal, Barcelona Centre for International Health Research (CRESIB) Hospital Clínic, Universitat de Barcelona, Barcelona, Spain; Médecins Sans Frontières (MSF), INDIA

## Abstract

**Background:**

Long-term success of HIV antiretroviral therapy requires near-perfect adherence, maintained throughout one’s lifetime. However, perceptions towards ART and patterns of adherence may change during the life course. We assessed challenges to long-term adherence in adolescents and adults in three regional HIV treatment centers in Uganda.

**Methods:**

We conducted 24 in-depth interviews and 2 focus group discussions with a total of 33 health-care providers and expert clients (HIV patients on long-term ART who assist with adherence support of fellow patients). Interview topics included experiences with patients on long-term treatment with either declining adherence or persistent poor adherence. Transcribed texts were coded and analyzed based on the social-ecological framework highlighting differences and commonalities between adolescents and adults.

**Results:**

The overarching themes in adolescents were unstructured treatment holidays, delays in disclosure of HIV status by caretakers, stigma, which was mainly experienced in boarding schools, and diminishing or lack of clinical support. In particular, there was minimal support for early and gradual disclosure for caretakers to the infected children, diminishing clinical support for young adults during transition to adult-based care and declining peer-to-peer support group activities. The predominating theme in adults was challenges with treatment access among temporary economic migrants. Common themes to adults and adolescents were challenges with disclosure in intimate relationships, treatment related factors including side effects, supply of single tablets in place of fixed-dose combined drugs, supply of drug brands with unfavorable taste and missed opportunities for counseling due to shortage of staff.

**Conclusion:**

Adherence counseling and support should be adapted differently for adolescents and adults and to the emerging life course challenges in long-term treated patients. Programs should also address constraints experienced by temporary economic migrants to ensure continuity of treatment within the host country.

## Introduction

Unprecedented scale-up of antiretroviral treatment (ART) in sub-Saharan Africa has remarkably changed the face of HIV epidemic from the previous fatal illness to a life-long chronic disease [1]. Subsequent sustenance of long-term and successful treatment outcomes depends primarily on achieving sustained viral suppression, supported by optimum life-long adherence [2]. Remarkably high levels of adherence have been reported in sub-Saharan Africa in comparison to western countries [3], but these findings contrast with those from other studies reporting increasing levels of patients with treatment failure [4,5], low retention [6] and high mortality rates [7]. Fewer studies have assessed adherence in long-term treated patients, despite the growing numbers as ART programs mature in the region [8–10].

Adherence over time may decline with duration of treatment [11,12] improve [8] or stabilize [13]. The reasons for decline in long-term treated patients may vary from those at the initial treatment phase [10,14]. As patient’s health improve, change in perception towards the disease, laxity to medication restrictions such as alcohol and tobacco use, non-disclosures in new sexual relationships and other life-course events may influence adherence to medication [10,14].

Of particular importance to long-term adherence is the emerging cohort of perinatally HIV infected children who are increasingly surviving into adolescence and adulthood. In 2014, about 2 million adolescents (aged 10–19) were living with HIV-AIDS (ALWHA), with 1.2 million (61%) in Eastern and Southern Africa [15]. ART adherence in ALWHA in sub-Saharan Africa is reported to be lower as compared to other age groups [16–18] including children [17,18] and declines also with increasing age [16,18]. Subsequently, adolescents have comparatively poor treatment responses [16,19] including high mortality [15,16].

Few studies have assessed long-term adherence among adolescents and young adults in sub-Saharan Africa [16,17,20–23]. A recent study in Uganda reported stigma, discrimination, attending rural health facilities and disclosure issues as main barriers to adherence [20]. Peer support groups, counseling, supportive health care workers, short waiting time, provision of food and transport were facilitators. More contextual data is needed to guide sustenance of long-term treatment adherence in this group. Although health-care workers play a pivotal role in providing adherence support and are thus usually more informed through their interactions with the patients and their understanding of the health-care system, few studies have included them when assessing adherence.

To conceptualize the barriers and facilitators to long-term ART adherence in both adolescents and adults, we conducted a qualitative study with health care workers and expert clients from three regional referral HIV treatment centers in Uganda.

## Methods

### Theoretical approach

This descriptive qualitative study draws from the social-ecological model (SEM) framework, which considers an individual’s behavior in this case ‘ART adherence’ as being a result of dynamic and complex interactions of factors at the various social-ecological levels in which the individual is situated [24]. An individual’s behavior is hence shaped based on the information, influence and interactions he/she obtains within one’s social-networks, social environment and institutions.

In sub-Saharan Africa, this includes cultural aspects, religious and society beliefs on the cause of the disease and its management [25]. In this way the construes of adherence do not only include the “how to adhere” as guided by the medication aspects and the “why to adhere” based on individual aspects but also the social component which influences the willingness and ability to adhere[25]. Indeed whether a person will adhere to treatment in such settings is heavily grounded in the social context and an individual must not only negotiate the how’s and why’s of adherence but also the societal positive and negative influences.

Moreover the individual is also dependent on the resources available to assist him/her to access and adhere to treatment [25–27]. In this way adherence to medication does not simply depends on the individual’s behavior but also upon structural factors within the individual social and environmental context.

In addition to social and structural factors, a patient’s adherence behavior is also influenced by program level factors. This includes such aspects as availability of drugs, distance and transport costs to ART clinics, quality of care, relationship with caregiver, as well as treatment factors such as dosing complexities[25–27].

### Setting

The study was conducted in three regional referral centers (RRCs) in Uganda; the Joint Clinical Research Center (JCRC) in Kampala and the RRCs in Fort Portal and Mbale, both run by the MOH. JCRC Kampala provides care to approximately 15,000 clients, 2,000 of whom are HIV-infected children and adolescents. RRC Mbale has approximately 4,200 patients on ART, with 400 children and adolescents; and RRC Fort Portal has approximately 7,474 patients, with approximately 500 children and adolescents. Ethical approval for this study was received from the Hospital Clinic-University of Barcelona Ethical Committee Board, the Joint Clinical Research Center ethical review board and the Uganda National Council for Science and Technology.

### Data collection

Data collection took place from May to August 2015, using semi-structured interviews (n = 24) with open-ended probes and focused group discussion (n = 2). We recruited participants based on purposive sampling so as to include more informative persons with regard to long-term adherence. In particular we included health-care workers who had longer years of experience with patients (Table 1), those specifically handling adolescents and adolescents transitioning to adult-based care (transitioners). We also included expert clients including adolescents themselves. Expert clients are HIV+ patients on long-term ART who assist the health-care workers mainly with adherence support of fellow patients. They play a vital role in bridging the gap caused by a limited health-care workforce but more important are able to reach out to patients at a more personal level based on their own experiences with the disease and treatment [28,29]. They provide counseling, motivate fellow patients by sharing their own personal experiences, conduct active tracing of patients lost-to-follow-up, offer adherence support and sometimes also assist health-care workers in minor clinical work such as in triage, patient flow and translation [28,29].

**Table 1:** Characteristics of study participants.

*Total participants*	*In-depth interview -N*	*FGDs-N*
Doctors	5	2
Nurses	11	6
Pharmacists	3	2
Counselors	9	6
Expert clients	5	1
*Characteristics*	*N*	
Years of experience with ART care and treatment (Median, IQR)	7 (2–23)	7 (4–8)
Female/male gender	22/11	14/3
*Site*	*N*	*N*
Joint clinical research center,	23	8
Mbale regional referral center	7	_
Fort Portal regional referral center	3	7

Data collection guidelines were designed to elicit health-care providers’ and expert clients’ views on declining adherence after long-term treatment and persistent poor adherence. Specifically the interview guides were set to elicit personal experiences of the health-care workers with poor-adherent patients and included the reasons given for the non-adherence as well as the management of these patients. The guides were also developed in such a way as to elicit broader aspects within the social-ecological framework, including personal-related barriers, social, and institutional-related barriers. In this way, the interviews provided deeper insights of both health-care workers personal experience with the clients as well as the broader systemic challenges known to impact on adherence. The guides were also adapted to gather the personal experiences of expert patients with ART adherence.

Through the study the guides were also progressively adapted within a *cyclic research design* where data gathered and analysed informed the next interviews. The iterative process was continued until saturation. Interviews were conducted at the clinic setting and lasted on average for 60–120 minutes. All the interviews were conducted in English by SCI; a junior research scientist-*MSc*, with close support from MR; a senior social scientist-*PhD*. All participants were provided with a detailed description of the study including the consent process and verbal consent was obtained and tape-recorded before each interview took place. The use of verbal consent was approved by the JCRC ethical review committee and was based on minimal risks associated with the study. All interviews were transcribed verbatim. Random fragments were sampled for quality checks and the data was imported and analyzed using the qualitative software package NVivo v.10.0 (QSR International). We employed a framework approach to analyze the data. We specifically used the social-ecological framework to create an initial coding guide that was also designed to highlight differences between adolescents and adults. This was done through discussions between SCI and MR. SCI then coded all the transcripts while MR checked the coded outputs. Codes were then revised and refined based on relevant and recurrent themes emerging from the data. Relationship codes were then created to analyze linkages and memos employed to document emerging themes. We then developed matrices to organize and iteratively compare the data indicating differences and commonalities between adolescents and adults.

## Results

A total of 33 individuals participated in this study including eleven nurses, nine adherence counselors, five medical doctors, five expert clients and three pharmacists (Table 1). Of these 24 participated in the interviews and 17 in the focus group discussions.

Eight key themes regarding long-term adherence were identified from the data, four of which were predominant among the adolescents; unstructured treatment holidays, delays in disclosure of HIV status to perinatally infected adolescents, stigma in boarding schools, and diminishing or lack of family and clinic support (extent that the clinic staff/structure helps the patient to adhere). In adults, challenge with treatment access among temporary economic migrants was the predominating theme. Common themes to adolescents and adults included disclosure in intimate relationships, treatment related factors and missed opportunities for counseling due to shortage of staff.

### Barriers specific to adolescents

#### Individual-level factors

**Unstructured treatment holidays** were noted amongst adolescents and were expressed as a desire to experience a drug-free life or a quest to understand the effects of being off drugs. However this was also as a result of underlying factors that included drug fatigue, pill burden, depression and stigma. Addressing these underlying factors could lead to resumption of treatment although in some cases the patients resumed treatment due to deteriorating health.

“There's a girl who…has been on treatment for some time but her viral load was high, so when I tried talking to her she was like "ah aah [No!]! Don’t tell me anything, I know everything, I’ve decided I’m not taking [the drugs]”.By the way she came today I was surprised!…when they asked her about her drugs she was like “I felt like I was tired of the drugs, so I needed a break”. Sometimes adolescents they give themselves breaks…for like 3 months but when he becomes sick, very sick, he comes back and says, “Now I want medicine”. It’s like they want to first see what will happen when [on] a break”.

**(Expert client-1)**

#### Social-level factors

**Delays in disclosing HIV status to perinatally infected children** prior to adolescence were common and could lead to non-adherence. In accordance with government regulation [30],it is the duty of the caretakers to disclose to their children, but they were reluctant and cited barriers such as fear of being blamed for the infection, fear of unintended disclosure of the parent HIV status by the child, fear of anticipated negative reactions and concern for lack of cognitive ability of the child to comprehend the implications of the disease on their health. Due to this, caretakers would sometimes lie to their children about their condition indicating that they were suffering from other chronic diseases like asthma or cancer. The concealment of status by implicating other diseases could however impact on the adherence of the adolescents as it foils the understanding of the importance of adherence and subsequent consequences, which are specific to HIV treatment. Moreover this could result in anger and depression when the adolescents become aware of their condition. Health-care providers could at times be obligated to assist with disclosure mainly when it was imperative for the child to know their status for example during adherence support at treatment failure or treatment switch which was done as a means of averting further failures.

“This time we had a very strict doctor whom they found [during clinic visit]…she said “You must disclose…because the boy is now 15 years and he is already on second-line [treatment]…So, the doctor took her [guardian] to the counselor by force, and they made her to disclose”

**(Pharmacist-1**)

Participants also noted that delays in disclosure were partly due to lack of supportive mechanisms to assist caretakers with disclosure.

“Caretakers can manage when they are prepared on time about disclosure. Usually they are stuck when you tell them [abruptly] that they need to tell the child. They are not prepared on how [to go about it]?”

**(Counselor-3)**

It was also noted that the lack of support for disclosure could result with poor or partial disclosures where children knew about their HIV status but did not fully comprehend the implications of the disease. Late or poorly disclosed adolescents were likely to stop treatment, react negatively to their caretakers and sometimes deny their HIV status.

**Diminishing or lack of family support:** Adolescents living with biological parents were perceived to have better adherence compared to those living with caretakers. The latter were reported to lack support for food, transport, medication reminders, and accompaniment by caretakers during clinic visits. Some adolescents also reported that the caretakers denied them education support on the misconception that they would die soon. However, as the adolescents grow older the need to be independent as well as other structural barriers could result with diminishing influence of the parents on the adolescent’s adherence behaviour or support.

“I’m asking [the mother] about so and so, she says "his drugs are at home" So the boy would take [a few] tin[s], if he is dispensed with 3-months supply on 2nd line, he would take 2 tins, leave the 4 at home. Then the mother would not bother, how comes, its long since he left his medicine. You would call her and she says "so and so took medicine, some is still at home". Till one time I told the woman that you carry all whatever you have at home [and bring to the clinic], she carried tins and tins and returned them”

**(Counselor- 1)**

**Perceived and experienced stigma in boarding schools:** Adherence among students in boarding schools was reported to be poor with the majority already being on second-line treatment. Poor adherence was linked to stigma, and this was influenced by where and how the students take their medication. Two groups emerged in the discussion: students who keep their own medication in the dormitories and those who keep them with the school nurse. Keeping drugs with the school nurse was reported to be beneficial due to likely reminders, close monitoring and easiness in getting permission for clinic visits. The preference to remain with the drugs was however due to having not disclosed to the school administration, the desire to be independent and fear of involuntary disclosure by the teachers or nurse to fellow students. Moreover, prevention messages such as ‘AIDS Kills’ placed in most schools also inadvertently stigmatized the infected students making it difficult for them to disclose.

Students keeping their own medication were said to face lack of privacy, anticipated (perceived) and enacted (experienced) stigma from peers and they also sometimes failed to access medication from the dormitories on time.

“There is a girl we lost, she passed away, she was 18… she had [experienced] stigma at school because they came across her drugs in her suitcase, and they pulled them out and they put them there and put her [medical] card on her bed and she was a head-girl and that killed her [spirit]! She had to switch school. Most of them you get these calls, when they are saying they have found out, you see, so she had to switch out schools”

**(Counselor-3)**

However, those keeping their medication with the school nurse also reported facing stigma emerging from their peers who persistently inquire of their frequent visits to the infirmary. Moreover these students could also experience stigma arising from inadvertent disclosures by the school nurse or administration.

“In the first school I disclosed…. but our nurse discriminated me…whenever the time of [taking] drugs approached she could come in the class [and say], "you don’t know that you have to take your drugs? Come and take your drugs" while all other students are there, [listening]… So they [other students] used to inquire what drugs do you take, the nurse is [always asking]…"you know you have your problem come and take your drugs ah"…If you don’t want you will die" ah, while other people are listening so [you get such] nicknames, “Mr. drugs, that is madam drugs”

**(Expert client-2)**

#### Health-care level factors

**Declining or lack of clinic support:** Most ALWHA at the study facilities were reported to have been infected through vertical transmission and had started ART early in life at the pediatric clinic. At 18 years of age they are expected to transition to the adult-based care. Transitioning was however a challenge due to difficulties in integrating with adult patients, reduced attention from health-care providers, long waiting hours, lack of medicine for treatment of other ailments and a general sense of abandonment from health care workers. Subsequently some of the adolescents would be lost-to-follow up.

“When we would transit them to adult [clinics], some of them would fail to come to pick their drugs, they were not getting the attention they were getting in the pediatric [section], because in pediatric, they are few [in numbers] and we try to know them personally”

**(Pharmacist-1)**

Although most participants expressed the importance of peer group support across all patients, it was noted that a decline in funding had either resulted with fewer activities or the complete phasing-out of these groups. The peer groups were reported to have served a pivotal role in providing an avenue for strengthening adherence, dealing with emerging challenges, providing motivation from peers and HIV-infected role models. Moreover, they also served as platform for social networking, where participants would get potential life partners. The subsequent decline of these groups was reported to be have impacted especially the adolescents with some of them opting out of care.

“Other challenges are the lack of peer groups and activities for adolescents. We used to have them but because of funding they stopped. And some adolescents dropped off after these activities and some even died. They had a strong belonging to these groups”

**(Counselor-6)**

Moreover, while it was noted that adolescents were a group in need of more counseling, this was hindered by a shortage of counselors. Frequent transfers of health-care providers due to shortage in staff also posed a problem to the adolescents who found it a challenge to confide and discuss freely about their health with new health-care providers. The use of social media coordinated by the clinic was a notably noble alternative, which was used both socially and formally to facilitate health-care providers and peer-peer counseling but was said to lack sufficient participation from health-care providers.

“And another thing is all about our social network pages, especially the Facebook ones. We need more counselors there…one can post via a friend so that others can know how to handle the problem but you fail to get even a counselor who can comment on the post or give advice on the post”

**(Expert client-2)**

### Barriers specific to adults

#### Individual-level factors

**Temporary migrants and challenges with treatment access:** Mobile persons especially those travelling temporarily to nearby countries for business comprised a substantial majority of patients and they had notably poor appointment keeping and drug pickups. Reasons given included lack of funds to cover for transport costs, perceived feeling of wellness hence prioritizing business to drug refill visits, desire to match clinic visits with trips for replenishing business stocks, and political conflicts in the host countries (mainly Southern Sudan and Congo), which affected their travel.

“I’ve had experiences of people saying that they travelled to Juba [South Sudan]…they go there for business…hoping to come back like after 3 months and the majority of them were complaining of this recent war that was in Juba, they could not make it…others talk about transport, they didn’t get enough money to come back [for drug refills] … then others talk of no proper health facility elsewhere that could give them drugs, otherwise they would have continued taking HAART…others would point-blank tell you they were not feeling ill, health-wise they were feeling ok, and since they were doing their business they decided to continue [with] their business”.

**(*Counselor-4)***

Other mobile populations included commercial sex workers, truck drivers, persons relocating to the villages for farming, and temporary emigrants. As a way of supporting these patients, they were supplied with drugs for 6–12 months instead of the custom 3 months so as to minimize on the number of drug-refill visits. Relatives or friends could also pick up drugs periodically on behalf of the patients but they would still be required to visit the clinics for blood tests at least once or twice a year. Despite these interventions, some clients still had poor appointment keeping prompting the health-care providers to encourage them to transfer to near-by facilities. Some were however reluctant to transfer-out citing poor standards in other facilities. Some migrants also reported reluctance by some of the host countries to provide them with ART.

### Common barriers

#### Individual-level factors

**Disclosure in intimate relationships:** New challenges with disclosure were cited when engaging in romantic relationships leading to compromises in adherence and potentially fuelling new infections. In adults, non-disclosure was linked to avoiding marital conflicts, fears of losing the partner or/and financial support. Adolescents generally lacked the skills to disclose and also feared losing their partner.

“In young adults, there are those getting boyfriends and girlfriends. They don’t disclose, and that is what prevents them from taking the drugs. They feel ashamed and don’t want to lose their partners”

**(Expert client-3)**

While support on disclosure was offered at the clinic, some health-care providers acknowledged their own bias when counseling discordant couples because they did not approve such relationships. This in turn could lead to the clients not to seek for disclosure support in subsequent times.

“They will tell you, when we take these partners to the hospital…. they are quick to judge you, you who is positive and yet you have brought in your partner really for testing. So they don’t help them and they end up breaking up instead. They don’t support them into this relationship and guide them on what to do. Instead they say heey! Trouble! So they separate them. So [they] are stuck with their relationships, they can’t come out open, [they have questions] who is going to support [me]… where should I go for counseling anyway? Because we are already biased”

**(Counselor-03)**

#### Health-care level factors

**Treatment-related factors:** Treatment side effects were also a concern for long-term treated patients in particular those arising after switch to second-line regimens. In adolescents this was said to result with re-emergence of stigma due to changes in body fat distribution from lopinavir-based treatment, and yellow eyes (jaundice) from atazanavir.

“We have many people who were changed to second-line, they were fat but now they are small. And they ask the doctor's why? Does ‘lopinavir’ takes off the kilograms…they fail to adhere whenever they see they have lost weight, they are looking bad, it seems as if ‘lopinavir’ is the cause”

**(Expert client-2)**

There were also challenges with drug supply logistics where separate pills were supplied for use by patients on fixed dose combinations (FDC). Moreover there were reports where facilities were supplied with pediatric formulations involving multiple low milligram tablets, for use by adults thereby increasing the pill burden.

“They send pediatric drugs for use in managing adults. So what we do, if the person has been taking like one pill, she will take 3 or 4, to make an adult dose. It is a challenge, which increases on client's pill burden”

**(Counselor-8)**

Moreover, adults also expressed a dislike of the sweet taste of pediatric drugs. In addition, facilities reported being supplied with drug of different brands, some that had bitter taste (uncoated drugs) and sometimes with varying colors and sizes. This was reported to confuse the patients and affect their adherence especially when they were given the bitter drugs. There were also claims of emerging side effects associated with change of drug brands.

“When we change from one company to another, some do experience some abnormal side-effects and they always say: “when I was taking the other type I didn’t have any problem but this time…”. Before we used to say that maybe they are used to this type of drug, but it does really affect them. When you change them and put them to a previous one, the complaints do go away.”

**(Pharmacist-2)**

Although there were no cases of drug stock-outs, there were reports of insufficient supply of drugs or those with shorter expiration period, which necessitated more frequent drug refill visits. There was also a limited supply of third-line drugs, which was mainly available to patients enrolled in medical research.

**Staff shortages and missed counseling opportunities:** Staff shortage leading to increased workload and long waiting times resulted in missed opportunities for adequately counseling the patients on vital information such as interpretation of laboratory test results. This could lead to misconceptions with some patients inferring that an undetectable viral load implied that they had been cured of the disease.

““So when [viral-load test] results were undetected, the person [clinician] would say that ‘akauka takalabika’ directly translating into the virus is invisible, so the patients would take it as gospel truth [or]…they say ‘akauka tekalio’, meaning the virus is not there…. The health worker should explain to those patients: last time we tested your viral load is undetectable, it doesn't mean you are healed, it simply means that you are taking your drugs well and the virus has just slept so you have to keep taking drugs so that the virus keeps sleeping. When you stop, it wakes up. But most of them don't get that explanation”.

**(FGD-2**)

## Discussion

Our study highlights pertinent insights into long-term ART adherence further distinguishing between adults and perinatally infected adolescents. In adults, a return to health with resumption of social and economic activities resulted in new dilemmas, potentially affecting their adherence. In adolescents, the challenges in adherence were mainly related to stigma, disclosure and declining clinic support.

### Mobility and adherence in adults

Emerging challenges to adherence with treatment progression in adults identified in previous studies suggest differences between early and long-term treated patients [10,14,31–33]. These studies have shown changes in disease perceptions, non-disclosure in intimate relationships, poor adherence to counselors’ instructions and re-emerging and persisting stigma as key challenges [10,14,31,33]. In our study, we further highlight challenges associated with mobile groups especially temporary business migrants as they strive for social and economic improvement. Although studies have documented the challenges faced by long-term migrants in accessing HIV care [34], there is still limited information on circular migrants, defined originally by Zelinsky as short-term, repetitive, or cyclical [35], which encompasses the temporary business migrants. The initial impediments to engagement in care and early adherence such as lack of transport and proper health care services [26,27] re-emerged in these long-term treated migrants, and were linked to a reluctance to transfer-out from their home facilities. In our study sites, preferential supply of treatment for longer duration as well as allowing treatment partners to pick-up drugs, were strategies that helped to maintain these patients on treatment. The preference to further transfer patients to closer facilities was however a challenge citing the reluctance by the host country to provide treatment to migrants. This is contrary to the recommendations by the ‘Global commission of HIV and the law’, directing that migrants be accorded equal treatment as citizens in matters relating to HIV[36].

### Stigma and ALWHA

As with previous studies, stigma was reported to impact on ART adherence especially among adolescents in boarding schools [20,23,37,38]. These findings corroborate other studies reporting the challenges faced by ALWHA in schools including discrimination from their peers and educators suggesting an increasing need for sensitization and training on their rights and special needs in school settings [23,38]. In addition, there is a need for a balanced approach in the design of HIV prevention messages in schools as the current ones such as ‘AIDS kills’ are discriminatory and further prevents ALWHA from disclosing and seeking support from either their peers or the school authorities.

Stigma among ALWHA was also associated with treatment side effects in particular visible body changes such as lipodystrophy [39] and ‘yellow eyes’ [40] from atazanavir. As adolescents are in particular careful about their physiques, it would be vital for treatment programs to consider implementing a personalized treatment management approach including switching to less toxic regimens once the patients have achieved viral suppression [41].

### Lack or declining clinic support for ALWHA

The pivotal role of health-care providers and the clinic environment to ALWHA is well known, with various studies showing the need for supportive early disclosure to caretakers [42–44], provision of adolescent friendly services [45], role of peer groups [20,45,46] and support for gradual transition to adult based care [45,46]. Despite this information, lack of clear guidelines and support was associated to late, incomplete and poor disclosures by caretakers and the subsequent decline in adherence [42]. Moreover, the need for gradual transition to adult-based care was a pertinent issue among adolescents in these facilities. Although guidance for transition exist in developed countries, there is little support for adaptation and implementation in sub-Saharan African settings [47]. Moreover the decline in funds leading to the phase-out of peer-peer support and the lack of adolescent-friendly counselors impacts negatively among the adolescents whose identity is strongly molded through the interactions within the clinic environment [48]. It is worth noting that most vertically infected adolescents are also orphaned and depend more on the close ties formed at the clinic for support and empowerment against the various challenges inherent in the community [23]. These findings continue to demonstrate the need for increased support of this group in the wake of increasing deaths of ALWHA in sub-Saharan Africa [16]. Alternative use of social media was seen as a platform to support adherence and cohesion among ALWHA. However, formal integration into existing health systems, coupled with sensitization and training of health-workers is needed to promote the use of these potential m-health tools in fostering adherence counseling in these settings.

### Challenges with drug supply logistics and influence on adherence

Apart from counseling, erratic drug supply logistics were also reported in these settings and in particular influenced adherence among patients on long-term treatment. This suggests the need for instituting quality systems to ensure continuous supply of appropriate regimen and formulations, prevent supply of uncoated bitter pills, and ensure sufficient communication to patients concerning the drug characteristics whenever there is a change in brand.

### Study limitations

There are some study limitations. First, we mainly included the views and experiences of health-care workers and expert clients and we may have missed other pertinent information from patients, including adolescents. The study had been specifically designed to address the barriers of long-term adherence based on the experience of health-care workers. The inclusion of health-care providers was based on the premise of the in-depth information they possess based on the nature of their interaction with HIV patients [49] and the additional advantage in their understanding of the health-care system. Our further inclusion of expert clients including adolescents provided crucial information to the study because of their a) Interactions with the patients at a more personal level: because of their own personal experiences with the disease and treatment, expert patients are viewed to be more relatable and empathetic to the challenges experience by the patients. This was vital among the adolescents who sometimes perceive the adult clinicians as being insensitive to their needs. The expert clients also have closer relationship with the patients, sometimes even visiting them at their homes to help address some of the difficulties they face. b) their own personal experience: In addition to the experience they have with the patients, the expert clients also shared their own experiences on the challenges they face with long-term ART adherence c) their view from both the perspective of the patient as well as from the health-care perspective: The information provided by the expert clients also reflects a balanced view of the barriers related to the health-care system owing to the fact that they serve as health-care workers and are also patients in the same facilities.

Second, our study was carried out in regional facilities and may underestimate the broader view especially from lower-level health facilities.

## Conclusion

Successful adherence among long-term treated patients in these setting is hampered by life-course events and is especially challenging among adolescents. There is thus need for programs to tailor adherence interventions to the emerging needs including support for disclosure to intimate partners and ways of ensuring continuity of treatment among temporary economic migrants. Moreover, programs should endeavor to support ALWHA in particular by offering support to caretakers for early and gradual disclosure of HIV status, supportive gradual transition to adult-based care as well as maintaining functional peer-support groups. Lastly there is need to train and sensitize educators and students against discrimination of ALWHA as well as empowering the infected children to cope with stigma in boarding schools.
