# Correction: Long-Term Antiretroviral Treatment Adherence in HIV-Infected Adolescents and Adults in Uganda: A Qualitative Study

**DOI:** 10.1371/journal.pone.0172077

**Published:** 2017-02-08

**Authors:** Seth C. Inzaule, Raph L. Hamers, Cissy Kityo, Tobias F. Rinke de Wit, Maria Roura

The second affiliation for the first author is not indicated. Seth C. Inzaule is also affiliated with: ISGlobal, Barcelona Centre for International Health Research (CRESIB) Hospital Clínic, Universitat de Barcelona, Barcelona, Spain.
